# Characteristics of Viral Shedding Time in SARS-CoV-2 Infections: A Systematic Review and Meta-Analysis

**DOI:** 10.3389/fpubh.2021.652842

**Published:** 2021-03-19

**Authors:** Danying Yan, Xiaobao Zhang, Can Chen, Daixi Jiang, Xiaoxiao Liu, Yuqing Zhou, Chenyang Huang, Yiyi Zhou, Zhou Guan, Cheng Ding, Lu Chen, Lei Lan, Xiaofang Fu, Jie Wu, Lanjuan Li, Shigui Yang

**Affiliations:** State Key Laboratory for Diagnosis and Treatment of Infectious Diseases, National Clinical Research Center for Infectious Diseases, Collaborative Innovation Center for Diagnosis and Treatment of Infectious Diseases, College of Medicine, The First Affiliated Hospital, Zhejiang University, Hangzhou, China

**Keywords:** viral shedding time, SARS- CoV-2, COVID-19, systematic review, meta-analysis

## Abstract

**Background:** The viral shedding time (VST) of SARS-CoV-2 mainly determines its transmission and duration of infectiousness. However, it was heterogeneous in the existing studies. Here, we performed a meta-analysis to comprehensively summarize the VST of SARS-CoV-2.

**Methods:** We searched PubMed, Web of Science, MedRxiv, BioRxiv, CNKI, CSTJ, and Wanfang up to October 25, 2020, for studies that reported VSTs of SARS-CoV-2. Pooled estimates and 95% CIs for the VSTs were calculated using log-transformed data. The VSTs in SARS-CoV-2 infections based on different demographic and clinical characteristics, treatments and specimens were stratified by subgroup analysis.

**Results:** A total of 35 studies involving 3,385 participants met the inclusion criteria. The pooled mean VST was 16.8 days (95% CI: 14.8–19.4, *I*^2^ = 99.56%) in SARS-CoV-2 infections. The VST was significantly longer in symptomatic infections (19.7 days, 95% CI: 17.2–22.7, *I*^2^ = 99.34%) than in asymptomatic infections (10.9 days, 95% CI: 8.3–14.3, *I*^2^ = 98.89%) (*P* < 0.05). The VST was 23.2 days (95% CI: 19.0–28.4, *I*^2^ = 99.24%) in adults, which was significantly longer than that in children (9.9 days, 95% CI: 8.1–12.2, *I*^2^ = 85.74%) (*P* < 0.05). The VST was significantly longer in persons with chronic diseases (24.2 days, 95% CI: 19.2–30.2, *I*^2^ = 84.07%) than in those without chronic diseases (11.5 days, 95% CI: 5.3–25.0, *I*^2^ = 82.11%) (*P* < 0.05). Persons receiving corticosteroid treatment (28.3 days, 95% CI: 25.6–31.2, *I*^2^ = 0.00%) had a longer VST than those without corticosteroid treatment (16.2 days, 95% CI: 11.5–22.5, *I*^2^ = 92.27%) (*P* = 0.06). The VST was significantly longer in stool specimens (30.3 days, 95% CI: 23.1–39.2, *I*^2^ = 92.09%) than in respiratory tract specimens (17.5 days, 95% CI: 14.9–20.6, *I*^2^ = 99.67%) (*P* < 0.05).

**Conclusions:** A longer VST was found in symptomatic infections, infected adults, persons with chronic diseases, and stool specimens.

## Introduction

Coronaviruses (CoVs), belonging to Nidovirales order, have caused three global outbreaks in the past 20 years. The first epidemic was Severe Acute Respiratory Syndrome (SARS) caused by SARS-CoV-1 in 2003, the second outbreak was Middle East Respiratory Syndrome (MERS) caused by MERS-CoV in 2012, and the third and most recent pandemic was Coronavirus Disease 2019 (COVID-19) caused by SARS-CoV-2 (1, 2). As of February 4, 2021, more than 104 million cases of COVID-19 have been reported with over 2.2 million deaths globally (3). Pulmonary clinical manifestations are the most common clinical presentations of COVID-19, such as fever, cough, shortness of breath, sputum production, respiratory failure and even acute respiratory distress syndrome (ARDS). Diarrhea, loss of smell or taste, and other extra-pulmonary clinical manifestations can also be found in some patients (4–7).

Persons infected with SARS-CoV-2 with long viral shedding times (VSTs) have drawn considerable concern, which put greater challenges and difficulties on epidemic prevention and control (8–11). The VST is an important parameter for judging hospital discharge, discontinuation of quarantine and the effect of antiviral treatment for infectious diseases, which mainly determines disease transmission and the duration of infectiousness (12). However, the characteristics of the VST in SARS-CoV-2 infections have not been well-clarified. Although there have been many studies on the VSTs of SARS-CoV-2, the results across studies so far have been heterogeneous (13, 14). A meta-analysis performed by Muge Cevik found that the mean VST of SARS-CoV-2 in the upper respiratory tract, lower respiratory tract, stool and serum was 17.0, 14.6, 17.2, and 16.6 days, respectively (15). However, a comprehensive summary of VSTs in SARS-CoV-2 infections with different demographic and clinical features is still lacking. Therefore, we performed a meta-analysis to estimate the mean VST in SARS-CoV-2 infections and explore the characteristics of VSTs in SARS-CoV-2 infections based on different demographic features, clinical characteristics, treatments and specimens.

## Materials and Methods

Our meta-analysis was strictly conducted in accordance with the Preferred Reporting Items for Systematic Reviews and Meta-Analyses Protocols (PRISMA-P) guidelines (16).

### The Definition of the VST

The definition of the VST varied among the studies, so a unified definition was made. We defined the VST as the time from illness onset to viral shedding cessation. Illness onset was defined as the first appearance of the symptoms for symptomatic infections and the first positive RT-PCR results for asymptomatic infections. Viral shedding cessation referred to the occurrence of the last positive RT-PCR results or negative RT-PCR results.

### Search Strategy and Selection Criteria

We searched PubMed, Web of Science, MedRxiv, BioRxiv, the China National Knowledge Infrastructure Database (CNKI), the China Science and Technology Journal Database (CSTJ), and the Wanfang Database up to October 25, 2020, for studies that reported VSTs of SARS-CoV-2. The details of the search strategy are shown in Supplementary Table 1.

In this systematic review with no study design limit, studies meeting the following inclusion criteria were eligible: (i) SARS-CoV-2 infections were based on positive RT-PCR results; (ii) the VSTs of SARS-CoV-2 infections including sample size, mean and standard deviation (SD) could be obtained directly from the original studies or by calculation; and (iii) the definition of the VST in the original studies was consistent with our definition.

We excluded (i) duplicated data; (ii) case reports and case series with <5 participants due to reporting bias; and (iii) studies without original data (e.g., modeling studies and reviews). Studies presenting VSTs with medians and interquartile ranges (IQRs) or ranges were excluded to reduce the errors caused by data conversion.

### Screening, Data Extraction, and Quality Assessment

After removing duplicates, two reviewers (DY and XZ) independently performed the initial screening of titles and abstracts to exclude studies that clearly contained no data for VST of SARS-CoV-2. All retained full-text articles were scrutinized against the eligibility criteria by two independent reviewers (DY and XZ). Nine investigators (DY, XZ, CC, DJ, XL, YZ, CH, YZ, and ZG) participated in the data extraction. And data extraction from each study was performed by three independent investigators. Disagreements and uncertainties were consulted by SY to reach a consensus. The following data were extracted: basic information of the studies (first author, publication time, journal name, sample size), VSTs in SARS-CoV-2 infections based on sex, age (adult and child), infection status (symptomatic infection and asymptomatic infection), disease severity (severe infection and non-severe infection), treatments (corticosteroid treatment and antiviral therapy) and specimens (respiratory tract specimens (RTS), upper respiratory tract specimens (URTS), lower respiratory tract specimens (LRTS), stool and serum). The cutoff point for classifying adults and children was 18 years old. Asymptomatic infections referred to the absence of any clinical symptoms throughout the disease course. Non-severe infections included mild and moderate infections, and severe infections included severe and critical infections. Antiviral drugs included interferon, lopinavir/ritonavir, abidor, ribavirin, chloroquine and hydroxychloroquine. URTS included nasopharyngeal, oropharyngeal and oronasopharyngeal swabs, and LRTS included sputum and bronchoalveolar lavage fluid.

The overall VST of the total participants was extracted to estimate the overall pooled VST. If studies did not report the overall VST of the total participants, the stratified VSTs were extracted to estimate the overall pooled VST. If a study included more than one independent study population, each population was extracted as a separate dataset in the meta-analysis. When the same study reported the VSTs of multiple specimens, the VSTs of the URTS were extracted to estimate the overall pooled VST, and the VSTs of other specimens were displayed in the subgroup analysis. In subgroup analysis, only studies having clear population characteristics were included in the corresponding subgroup, and studies having no clear information or mixed population group were included in the unclassified group.

The scale recommended by the Agency for Healthcare Research and Quality was used to assess the quality of the included studies (17). The scale consists of 11 items, and 1 point is given to each item when the conditions are met. It mainly focuses on information source, inclusion and exclusion criteria, study period, selection of participants, evaluation of subjective outcomes/components, quality assurance, possible confounding variables, handling of missing data, participants' response rates and completeness of data collection. According to the total score, the studies were divided into low-(0–3), medium-(4–7) and high-quality (8–11) groups. EndNote (version X9) was used to manage the articles and citations.

### Statistical Analysis

We first extracted the individual VSTs from the published articles and found that the distribution type of the VST was approximately in accordance with the log-normal distribution by using P-P plots (Supplementary Figure 1). Then, we used the method developed by McAloon C (18) to transform the original VST data to make the data obey a normal distribution. We used random-effects model to perform the meta-analysis due to the high heterogeneity. Finally, we used the method developed by McAloon C (18) to back-transform the point estimates and their 95% confidence intervals (CIs). The *I*-squared (*I*^2^) statistic was used to evaluate the heterogeneity among the studies. Meta-regression was used to quantify the sources of heterogeneity and to explore the level of significance between subgroup comparisons. We did not assess publication bias because usual appraisal methods are uninformative when studies in the meta-analysis do not include a test of significance. The data cleaning and analysis were performed using the Microsoft Excel 2016 and R version 3.2.3.

## Results

**Figure 1:**
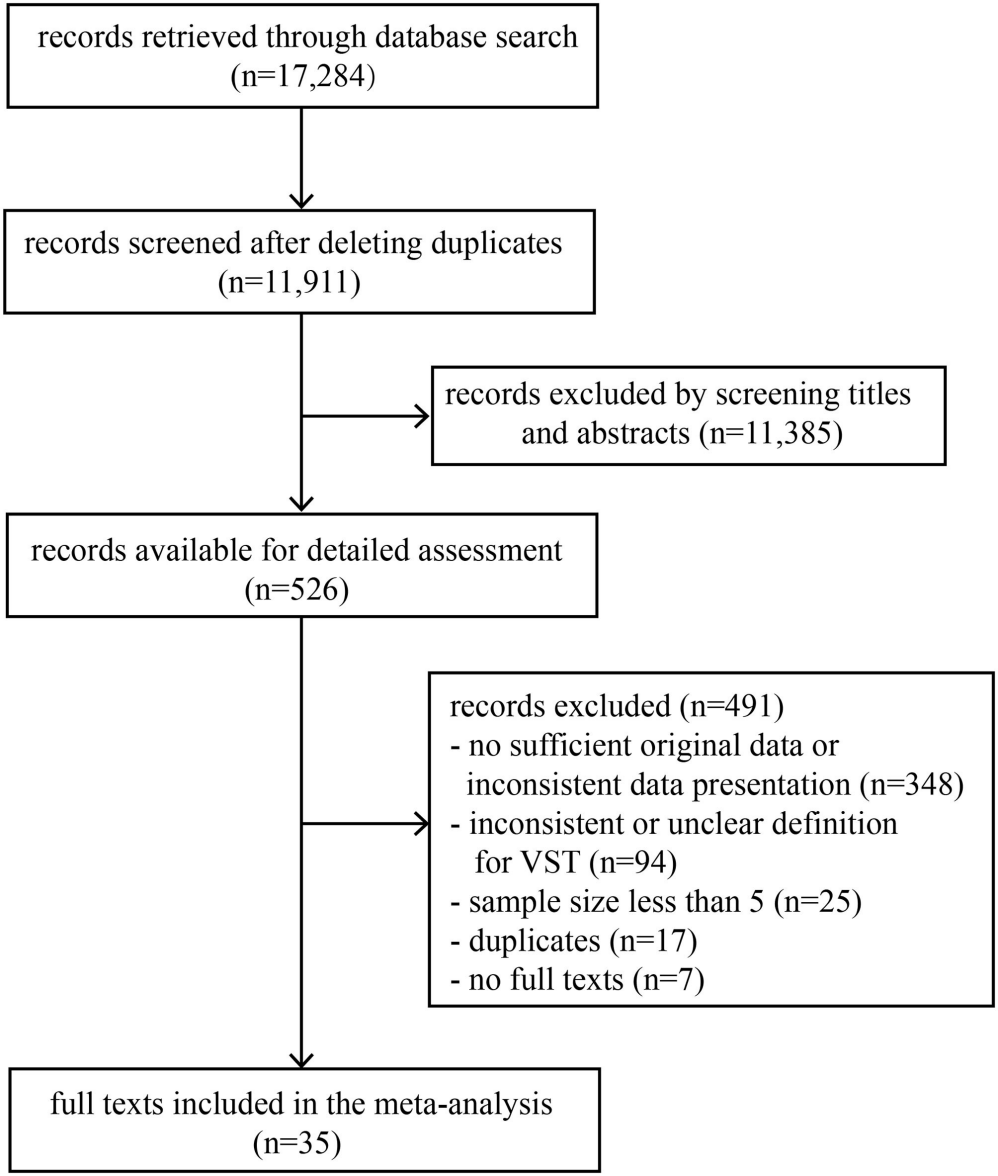
Flow diagram of the study selection process for this meta-analysis.

A total of 17,284 records were retrieved through a database search. The titles and abstracts of 11,911 records were screened after deleting duplicates, and then 526 records were selected for full-text review. Finally, 35 full texts met the inclusion criteria (Figure 1). This study included 35 observational studies and involved 3,385 individuals infected with SARS-CoV-2, of which 2,955 were symptomatic infections and 338 were asymptomatic infections (Table 1). According to the scale, 32 studies were of high quality, 3 studies were of medium quality and none were of low quality (Table 1 and Supplementary Table 2).

**Table 1:** Characteristics of included studies.

**References**	**Country**	**Study design**	**Sample size, n**	Age, years^*^	**Female, n (%)**	**Infection status**	**Asymptomatic case^**#**^, n (%)**	**Specimen types**	**Study quality**
Jiehao et al. (19)	China	Case series	10	6.5	6 (60)	sym	0 (0)	URTS	8 (High)
Xiong et al. (20)	China	Cohort study	51	/	21 (41)	asym	51 (100)	URTS	8 (High)
Yang et al. (21)	China	Case series	5	49	2 (40)	sym, asym	2 (40)	URTS	8 (High)
Noh et al. (22)	Korea	Cohort study	53	/	/	asym	53 (100)	/	8 (High)
Zheng et al. (23)	China	Cohort study	1,320	50	741 (56)	sym	0 (0)	URTS	8 (High)
Lee et al. (24)	Korea	Cohort study	89	22	55 (62)	asym	89 (100)	RTS	8 (High)
Song et al. (25)	China	Case series	16	8.5	6 (38)	sym, asym	8 (50)	URTS	8 (High)
Jun et al. (26)	China	Cross-sectional study	242	/	/	sym	0 (0)	URTS	8 (High)
Zhu et al. (27)	China	Case series	20	/	/	sym	0 (0)	URTS	8 (High)
Han et al. (28)	China	Cohort study	206	62.5	115 (56)	sym	0 (0)	URTS	8 (High)
Yan et al. (29)	China	Cross-sectional study	24	/	/	asym	24 (100)	RTS	8 (High)
Gong et al. (30)	China	Cohort study	34	/	12 (35)	sym	0 (0)	URTS	8 (High)
Warabi et al. (31)	Japan	Cross-sectional study	8	14	6 (75)	sym	0 (0)	URTS	8 (High)
Pan et al. (32)	China	Cross-sectional study	26	29.5	10 (38)	asym	26 (100)	RTS	7 (Medium)
Hua et al. (33)	China	Cross-sectional study	43	/	/	/	/	URTS	8 (High)
Cano et al. (34)	Switzerland	Cohort study	251	53	103 (41)	sym	0 (0)	URTS	7 (Medium)
Wu et al. (35)	China	Cross-sectional study	74	/	/	sym	0 (0)	URTS, stool	8 (High)
Otsubo et al. (36)	/	Case series	5	74	2 (40)	sym	0 (0)	URTS	7 (Medium)
Tan et al. (37)	China	Case series	12	34.5	3 (25)	asym	12 (100)	URTS	8 (High)
Xiao et al. (38)	China	Cohort study	63	/	/	sym, asym	19 (30)	/	8 (High)
Yao et al. (39)	China	Case series	5	47	3 (60)	sym, asym	1 (20)	URTS	8 (High)
Liu et al. (40)	China	Cohort study	53	8	19 (36)	asym	53 (100)	URTS	8 (High)
Shi et al. (41)	China	Cross-sectional study	33	41	14 (42)	sym	0 (0)	URTS	8 (High)
Li et al. (42)	China	Cohort study	46	45.6	25 (54)	sym	0 (0)	/	8 (High)
Jiang et al. (43)	China	Cross-sectional study	24	37	10 (42)	sym	0 (0)	/	8 (High)
Gong et al. (44)	China	Cross-sectional study	179	57.4	90 (50)	sym	0 (0)	URTS	8 (High)
Zhao et al. (45)	China	Cohort study	63	/	32 (51)	sym	0 (0)	/	8 (High)
Zhang et al. (46)	China	Cross-sectional study	30	/	/	sym	0 (0)	RTS, stool	8 (High)
Xu et al. (47)	China	Cohort study	59	49.3	31 (53)	sym	0 (0)	URTS	8 (High)
Xie et al. (48)	China	Cross-sectional study	49	49.4	24 (49)	/	/	/	8 (High)
Sun et al. (49)	China	Cross-sectional study	46	/	/	sym	0 (0)	RTS	8 (High)
Ren et al. (50)	China	Cross-sectional study	89	/	/	sym	0 (0)	RTS	8 (High)
Ran et al. (51)	China	Cross-sectional study	28	59.4	9 (32)	sym	0 (0)	RTS	8 (High)
Liu et al. (53)	China	Cross-sectional study	41	68	21 (51)	sym	0 (0)	URTS	8 (High)
Li et al. (54)	China	Cohort study	88	46	34 (39)	sym	0 (0)	URTS	8 (High)

*sym, symptomatic infection; asym, asymptomatic infection; RTS, respiratory tract specimen; URTS, upper respiratory tract specimen*.

* : Median or mean;

# *: Asymptomatic cases with VST of SARS-CoV-2; /: Unreported or unclassified or incalculable*.

### VSTs in SARS-CoV-2 Infections and Subgroup Results Based on Clinical Characteristics

**Figure 2:**
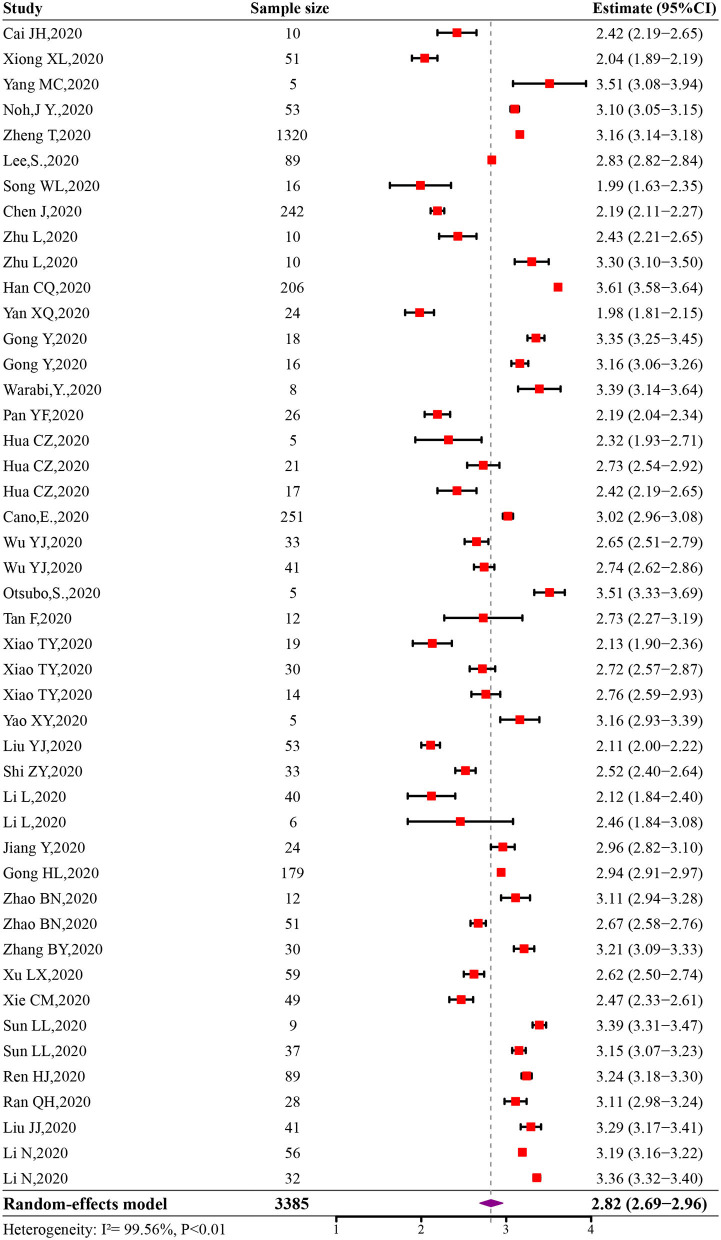
Forest plot of meta-analysis of log-transformed VST in SARS-CoV-2 infections.

**Figure 3:**
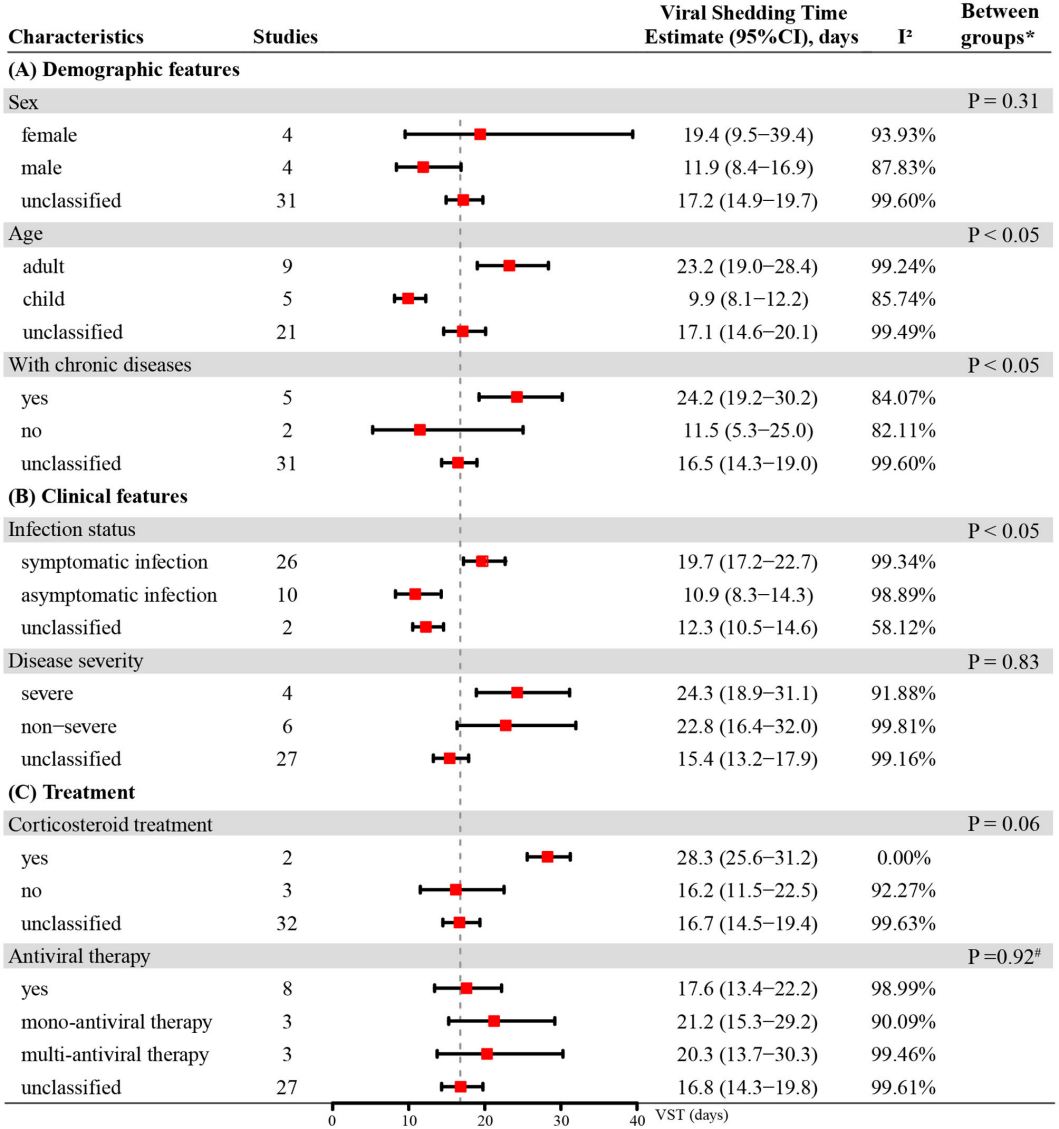
The VST in SARS-CoV-2 infections based on different demographic features, clinical features and treatments. * Unclassified groups were not included in the *P*-values calculation for the subgroup comparisons. #*P*-value for comparison between group with antiviral mono-therapy and group with antiviral muti-therapy.

The initial pooled estimate of the log-transformed VST in SARS-CoV-2 infections was 2.82 (95% CI: 2.69–2.96) (Figure 2). The pooled mean VST was 16.8 days (95% CI: 14.8–19.4) in SARS-CoV-2 infections. The mean VST of symptomatic infections was 19.7 days (95% CI: 17.2–22.7), which was significantly longer than that of asymptomatic infections (10.9 days, 95% CI: 8.3–14.3) (*P* < 0.05). The mean VST was 24.3 days (95% CI: 18.9–31.1) in severe patients and 22.8 days (95% CI: 16.4–32.0) in non-severe patients (Figure 3).

### VSTs in SARS-CoV-2 Infections Subgrouped by Demographic Features

The mean VST was 19.4 days (95% CI: 9.5–39.4) in females and 11.9 days (95% CI: 8.4–16.9) in males. The VST was significantly shorter in the infected children (9.9 days, 95% CI: 8.1–12.2) than in the infected adults (23.2 days, 95% CI: 19.0–28.4) (*P* < 0.05). The VST of persons with chronic diseases was 24.2 days (95% CI: 19.2–30.2), which was significantly longer than that of persons without chronic diseases (11.5 days, 95% CI: 5.3–25.0) (*P* < 0.05) (Figure 3).

### VSTs in SARS-CoV-2 Infections Subgrouped by Treatments

In persons receiving corticosteroid treatment, the VST was 28.3 days (95% CI: 25.6–31.2), which was longer than that in those without corticosteroid treatment (16.2 days, 95% CI: 11.5–22.5). However, there was no statistically significant difference between them (*P* = 0.06). The VST was 17.6 days (95% CI: 13.4–22.2) in persons receiving antiviral therapy, 21.2 days (95% CI: 15.3–29.2) in persons receiving mono-antiviral therapy and 20.3 days (95% CI: 13.7–30.3) in persons receiving multi-antiviral therapy (Figure 3). Only one study reported the VSTs of 5 patients without antiviral therapy, and the result was 11.2 ± 5.2 days (33).

### VSTs in SARS-CoV-2 Infections Subgrouped by Different Specimens

**Figure 4:**
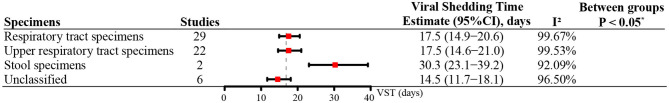
The VST in SARS-CoV-2 infections based on different specimens. *P-value for comparison between respiratory tract specimens and stool specimens.

Most studies (63%) reported the VSTs in the URTS. Among the different specimens, the mean VST was 17.5 days (95% CI: 14.9–20.6) in the RTS and 17.5 days (95% CI: 14.6–21.0) in the URTS. Compared with the RTS, a longer VST was found in the stool specimens (30.3 days, 95% CI: 23.1–39.2) (*P* < 0.05) (Figure 4). No included study reported VSTs in LRTS or serum specimens.

### Meta-Regression for Heterogeneity

The univariate meta-regression model indicated that the mean age (*R*^2^ = 35.28%, *P* < 0.05) and the proportion of the asymptomatic cases (*R*^2^ = 22.64%, *P* < 0.05) could partly explain the overall heterogeneity. By introducing these two variables into the multivariate meta-regression model, nearly half of the heterogeneity could be explained (*R*^2^= 44.18%, *P* < 0.05) (Supplementary Table 3).

## Discussion

We performed a meta-analysis to clarify the characteristics of VSTs in SARS-CoV-2 infections, which was important for determining hospital discharge, discontinuation of quarantine and the effect of antiviral treatment for COVID-19. Compared with the meta-analysis conducted by Muge Cevik (15), our study not only estimated the VSTs in different specimens but also summarized the VSTs in SARS-CoV-2 infections based on different demographic features, clinical characteristics and treatments.

Previous studies have shown that the basic reproduction number (R0) of SARS-CoV-2 is between 2 and 6.7, which indicates that SARS-CoV-2 is more infectious than SARS-CoV-1 and MERS-CoV (55–57). In our study, we found that the mean VST of SARS-CoV-2 was 16.8 days (95% CI: 14.8–19.4), which was between that of SARS-CoV-1 (21.0 days) and MERS-CoV (13.2 days) (58, 59). In addition to the VST, the viral load released is also important to evaluate the transmissibility. Some studies have found that the viral load of SARS-CoV-2 is highest during the 1st week after symptom onset and subsequently declines with time (60–62). Based on the above analysis, from the perspective of epidemic prevention and control, strict precautions should be taken throughout the disease course, especially within 1 week after the onset of the disease.

The duration of viral shedding is mainly related to the host immune status (63). Persons with chronic diseases always have relatively low immunity, which might lead to longer viral shedding. In our study, we found that the VST of symptomatic infections was longer than that of asymptomatic infections. One reason is that virus clearance in asymptomatic individuals is indeed faster than that in symptomatic cases (38, 52). Another reason was that the VST for asymptomatic infections was calculated from the first positive PCR results and depended mainly on close contact tracking investigations. These individuals might have begun viral shedding before the first positive PCR results, and were ignored due to the absence of clinical features. A higher proportion of asymptomatic infections and milder clinical symptoms were found in infected children compared with infected adults (64, 65), which might explain the shorter VST of children.

The VST is an important parameter for evaluating the effect of antiviral treatment for infectious diseases. Until now, there have been no specific antiviral drugs for COVID-19, and inhibiting the cytokine storm has been an important treatment for patients with severe COVID-19. Corticosteroids are used because of their rapid, powerful anti-inflammatory effects. In our study, we found that the patients who received corticosteroid treatment had longer VSTs, although no statistically significant difference was found. This phenomenon was also found in severe SARS and MERS, where high-dose corticosteroids could cause prolonged viral clearance, secondary infection and long-term complications (66). Although corticosteroids can inhibit lung inflammation and alleviate possible immune-mediated pulmonary damage, it can also inhibit the systemic immune response dominated by T cell response, resulting in the delayed virus clearance (67). This finding alerted us that high-dose corticosteroids might prolong VSTs in SARS-CoV-2 infections and that appropriate doses of corticosteroids should be used after weighing the advantages and disadvantages according to the patients' condition.

The VST is also an important parameter for determining hospital discharge and discontinuation of quarantine. Two consecutive negative PCR results of RTS are one of the current criteria for hospital discharge or discontinuation of quarantine in China (68). The overexpression of ACE-2 in the gastrointestinal (GI) epithelial cells suggested the replication and shedding of SARS-CoV-2 in GI tract (69). Similar to SARS-CoV-1 (59), the VST of SARS-CoV-2 in stool specimens was longer than that in RTS. One study suggested that the VST in stool specimens could be prolonged by 5 weeks after SARS-CoV-2 had turned negative in RTS (35). Given that, the negative PCR results in RTS might not guarantee that patients no longer shed virus. Recently, several incidents of cold chain food polluted by SARS-CoV-2 have caused widespread concern by indicating that the virus could indeed infect individuals by polluting the environment. Considering the potential risk of oral-fecal transmission (70) and the long VST in stool specimens, more comprehensive protective measures should be taken for high-risk groups of oral-fecal transmission, such as GI endoscopy staff (2, 71), and stool or anal swabs collection and testing staff.

Our results might provide scientific support for the formulation of antiviral treatment and criteria for hospital discharge and discontinuation of quarantine for COVID-19, and help identify which patients need more attention and more effective preventive measures. Based on the mean VST of SARS-CoV-2 infections, hospitals could estimate the number of individuals with COVID-19 who can be admitted in a period of time, and reasonably allocate medical resources, such as the number of beds and medical staff.

This study has several limitations. The mean age, disease severity, treatment regimens, underlying diseases and infection status of individuals infected with SARS-CoV-2 varied in the included studies, which might cause high statistical heterogeneity. In the multivariate meta-regression model, nearly half of the heterogeneity could be explained by mean age and the proportion of the asymptomatic cases (*R*^2^= 44.18%, *P* < 0.05). In some subgroup analyses, the number of included studies was small and most were case series with limited sample sizes, which might make the effect size of some outcomes insufficient. For example, the pooled mean VST in the stool specimens was based on estimates obtained in only two studies. More studies on the VST of SARS-CoV-2 are needed to provide further evidence. It would be better to incorporate as many studies as possible to obtain sufficient subgroup data and to ensure the homogeneity of the studies. Furthermore, the day of symptom onset for symptomatic infections depended on subjective memories and the day of the first positive RT-PCR results for asymptomatic infections relied mainly on close contact tracking investigations. If the individuals' recall was incorrect or close contact tracking investigations were not timely, these would cause the obtained VSTs to deviate from the real values.

## Conclusions

This study provided a comprehensive overview of VSTs in SARS-CoV-2 infections, which was important for determining hospital discharge, discontinuation of quarantine and the effect of antiviral treatment for COVID-19. The pooled mean VST was 16.8 days (95% CI: 14.8–19.4) in SARS-CoV-2 infections. Due to the high infectivity of SARS-CoV-2, strict precautions should be taken to reduce the risk of disease transmission, especially for adults, persons with chronic diseases, symptomatic infections and persons with positive RT-PCR results in stool specimens, in whom longer VSTs were found. Given that high-dose corticosteroids could alleviate possible immune-mediated pulmonary damage but might prolong VSTs in SARS-CoV-2 infections, corticosteroids should be used with caution after analyzing the risk of prolonged VST with reducing the disease severity.

## Data Availability Statement

The original contributions presented in the study are included in the article/Supplementary Material, further inquiries can be directed to the corresponding author/s.

## Author Contributions

SY, LJL, and JW designed the study and revised the manuscript. DY and XZ independently performed the literature identification and uncertainties were consulted by SY to reach a consensus. DY, XZ, CC, DJ, XL, YZ, CH, YZ, and ZG extracted data. CD, LC, LL, and XF rechecked the data. DY carried out the data analysis. DY and XZ interpreted data and wrote the manuscript. All authors have read and approved the final version of the manuscript for submission.

## Conflict of Interest

The authors declare that the research was conducted in the absence of any commercial or financial relationships that could be construed as a potential conflict of interest.
