# Supplementary material for: Characteristics of Viral Shedding Time in SARS-CoV-2 Infections: A Systematic Review and Meta-Analysis
Source: Front Public Health. 2021 Mar 19;9:652842. doi: 10.3389/fpubh.2021.652842 (PMC8017277; doi:10.3389/fpubh.2021.652842)
Supplement: Supplementary file 1 [file Data_Sheet_1.pdf]

Supplementary table 1. The search strategy of viral shedding time of SARS-CoV-2*

Database	Searches
Pubmed	(((((((severe acute respiratory syndrome coronavirus 2[Supplementary Concept]) OR COVID-19[Supplementary Concept]) OR COVID-19[Text Word]) OR SARS-CoV-2[Text Word]) OR coronavirus disease 2019[Text Word]) OR novel coronavirus[Text Word]) OR coronavirus[Text Word]) AND (((((((shed*[Text Word]) OR (conversion[Text Word])) OR (clearance[Text Word])) OR (carr*[Text Word])) OR (communicable[Text Word])) OR (excret*[Text Word])) OR (secret*[Text Word])) Filters: from 2020 - 2020
Web of Science	TS: ((((((((severe acute respiratory syndrome coronavirus 2) OR COVID-19) OR SARS-CoV-2) OR coronavirus disease 2019) OR novel coronavirus) OR coronavirus))) AND TS: (((((((shed*) OR (conversion)) OR (clearance)) OR (carr*)) OR (communicable)) OR (excret*)) OR (secret*)) Filters: from 2020 - 2020
MedRxiv	(COVID-19 OR 2019-nCoV OR SARS-CoV-2) AND (shed OR conversion OR clearance OR carriage OR communicable OR excrete OR secrete) Filters: 2020/01/01-2020/10/25
BioRxiv	(COVID-19 OR 2019-nCoV OR SARS-CoV-2) AND (shed OR conversion OR clearance OR carriage OR communicable OR excrete OR secrete) Filters: 2020/01/01-2020/10/25

* Only the search strategies of English databases were shown.

Supplementary table 2. The quality of the included studies

First Author, Year	Item 1	Item 2	Item 3	Item 4	Item 5	Item 6	Item 7	Item 8	Item 9	Item 10	Item 11	Total	Quality
Cai JH,2020(1)	1	1	1	1	1	0	1	0	0	1	1	8	High
Xiong XL,2020(2)	1	1	1	1	1	0	1	0	0	1	1	8	High
Yang MC,2020(3)	1	1	1	1	1	0	1	0	0	1	1	8	High
Noh, J.Y.,2020(4)	1	1	1	1	1	0	1	0	0	1	1	8	High
Zheng T,2020(5)	1	1	1	1	1	0	1	0	0	1	1	8	High
Lee, S.,2020(6)	1	1	1	1	1	0	1	0	0	1	1	8	High
Song WL,2020(7)	1	1	1	1	1	0	1	0	0	1	1	8	High
Chen J,2020(8)	1	1	1	1	1	0	1	0	0	1	1	8	High
Zhu L,2020(9)	1	1	1	1	1	0	1	0	0	1	1	8	High
Han CQ,2020(10)	1	1	1	1	1	0	1	0	0	1	1	8	High
Yan XQ,2020(11)	1	1	1	1	1	0	1	0	0	1	1	8	High
Gong Y,2020(12)	1	1	1	1	1	0	1	0	0	1	1	8	High
Warabi, Y.,2020(13)	1	1	1	1	1	0	1	0	0	1	1	8	High
Pan YF,2020(14)	1	1	0	1	1	0	1	0	0	1	1	7	Medium
Hua CZ,2020(15)	1	1	1	1	1	0	1	0	0	1	1	8	High
Cano, E.,2020(16)	1	1	0	1	1	0	1	0	0	1	1	7	Medium
Wu YJ,2020(17)	1	1	1	1	1	0	1	0	0	1	1	8	High
Otsubo, S.,2020(18)	1	1	0	1	1	0	1	0	0	1	1	7	Medium
Tan F,2020(19)	1	1	1	1	1	0	1	0	0	1	1	8	High
Xiao TY,2020(20)	1	1	1	1	1	0	1	0	0	1	1	8	High
Yao XY,2020(21)	1	1	1	1	1	0	1	0	0	1	1	8	High
Liu YJ,2020(22)	1	1	1	1	1	0	1	0	0	1	1	8	High
Shi ZY,2020(23)	1	1	1	1	1	0	1	0	0	1	1	8	High

Li L,2020(24)	1	1	1	1	1	0	1	0	0	1	1	8	High
Jiang Y,2020(25)	1	1	1	1	1	0	1	0	0	1	1	8	High
Gong HL,2020(26)	1	1	1	1	1	0	1	0	0	1	1	8	High
Zhao BN,2020(27)	1	1	1	1	1	0	1	0	0	1	1	8	High
Zhang BY,2020(28)	1	1	1	1	1	0	1	0	0	1	1	8	High
Xu LX,2020(29)	1	1	1	1	1	0	1	0	0	1	1	8	High
Xie CM,2020(30)	1	1	1	1	1	0	1	0	0	1	1	8	High
Sun LL,2020(31)	1	1	1	1	1	0	1	0	0	1	1	8	High
Ren HJ,2020(32)	1	1	1	1	1	0	1	0	0	1	1	8	High
Ran QH,2020(33)	1	1	1	1	1	0	1	0	0	1	1	8	High
Liu JJ,2020(34)	1	1	1	1	1	0	1	0	0	1	1	8	High
Li N,2020(35)	1	1	1	1	1	0	1	0	0	1	1	8	High

Item 1: Define the source of information (survey, record review); Item 2: List inclusion and exclusion criteria for exposed and unexposed subjects (cases and controls) or refer to previous publications; Item 3: Indicate time period used for identifying patients; Item 4: Indicate whether or not subjects were consecutive if not population-based; Item 5: Indicate if evaluators of subjective components of study were masked to other aspects of the status of the participants; Item 6: Describe any assessments undertaken for quality assurance purposes (e.g., test/retest of primary outcome measurements); Item 7: Explain any patient exclusions from analysis; Item 8: Describe how confounding was assessed and/or controlled; Item 9: If applicable, explain how missing data were handled in the analysis; Item 10: Summarize patient response rates and completeness of data collection; Item 11: Clarify what follow-up, if any, was expected and the percentage of patients for which incomplete data or follow-up was obtained.

Supplementary table 3. Meta-regression for heterogeneity

Mean age

Mixed-Effects Model (k = 26; tau^2 estimator: REML)	
tau^2 (estimated amount of residual heterogeneity):	0.1284 (SE = 0.0379)
tau (square root of estimated tau^2 value):	0.3583
I^2 (residual heterogeneity / unaccounted variability):	99.78%
H^2 (unaccounted variability / sampling variability):	460.16
R^2 (amount of heterogeneity accounted for):	35.28%
Test for Residual Heterogeneity:	
QE(df = 24) = 4822.2862, p-val < .0001	
Test of Moderators (coefficient 2):	
QM(df = 1) = 14.3224, p-val = 0.0002	

Model Results:

	estimate	se	zval	pval	ci.lb	ci.ub	
intrcpt	2.3082	0.1812	12.7369	<.0001	1.9530	2.6634	***
age	0.015	0.004	3.7845	0.0002	0.0072	0.0228	***

Signif. codes: 0 ‘***’ 0.001 ‘**’ 0.01 ‘*’ 0.05 ‘.’ 0.1 ‘ ’ 1

The proportion of the asymptomatic cases

Mixed-Effects Model (k = 42; tau^2 estimator: REML)	
tau^2 (estimated amount of residual heterogeneity):	0.1737 (SE = 0.0395)
tau (square root of estimated tau^2 value):	0.4167
I^2 (residual heterogeneity / unaccounted variability):	99.78%
H^2 (unaccounted variability / sampling variability):	461.58
R^2 (amount of heterogeneity accounted for):	22.64%
Test for Residual Heterogeneity:	
QE(df = 40) = 9227.5981, p-val < .0001	
Test of Moderators (coefficient 2):	
QM(df = 1) = 12.5236, p-val = 0.0004	

Model Results:

	estimate	se	zval	pval	ci.lb	ci.ub	
intrcpt	2.9743	0.074	40.1947	<.0001	2.8292	3.1193	***
asym	-0.0059	0.0017	-3.5389	0.0004	-0.0091	-0.0026	***

Signif. codes: 0 ‘***’ 0.001 ‘**’ 0.01 ‘*’ 0.05 ‘.’ 0.1 ‘ ’ 1

Mean age and the proportion of the asymptomatic cases

Mixed-Effects Model (k = 25; tau^2 estimator: REML)	
tau^2 (estimated amount of residual heterogeneity):	0.1100 (SE = 0.0340)
tau (square root of estimated tau^2 value):	0.3317
I^2 (residual heterogeneity / unaccounted variability):	99.72%
H^2 (unaccounted variability / sampling variability):	360.21
R^2 (amount of heterogeneity accounted for):	44.18%
Test for Residual Heterogeneity:	
QE(df = 22) = 4524.7241, p-val < .0001	

Test of Moderators (coefficients 2:3):

QM(df = 2) = 20.3544, p-val < .0001

Model Results:

	estimate	se	zval	pval	ci.lb	ci.ub	
intrcpt	2.5262	0.2148	11.7619	<.0001	2.1053	2.9472	***
age	0.012	0.0043	2.7932	0.0052	0.0036	0.0204	**
asym	-0.0035	0.0021	-1.6235	0.1045	-0.0076	0.0007	

Signif. codes: 0 ‘***’ 0.001 ‘**’ 0.01 ‘*’ 0.05 ‘.’ 0.1 ‘ ’ 1

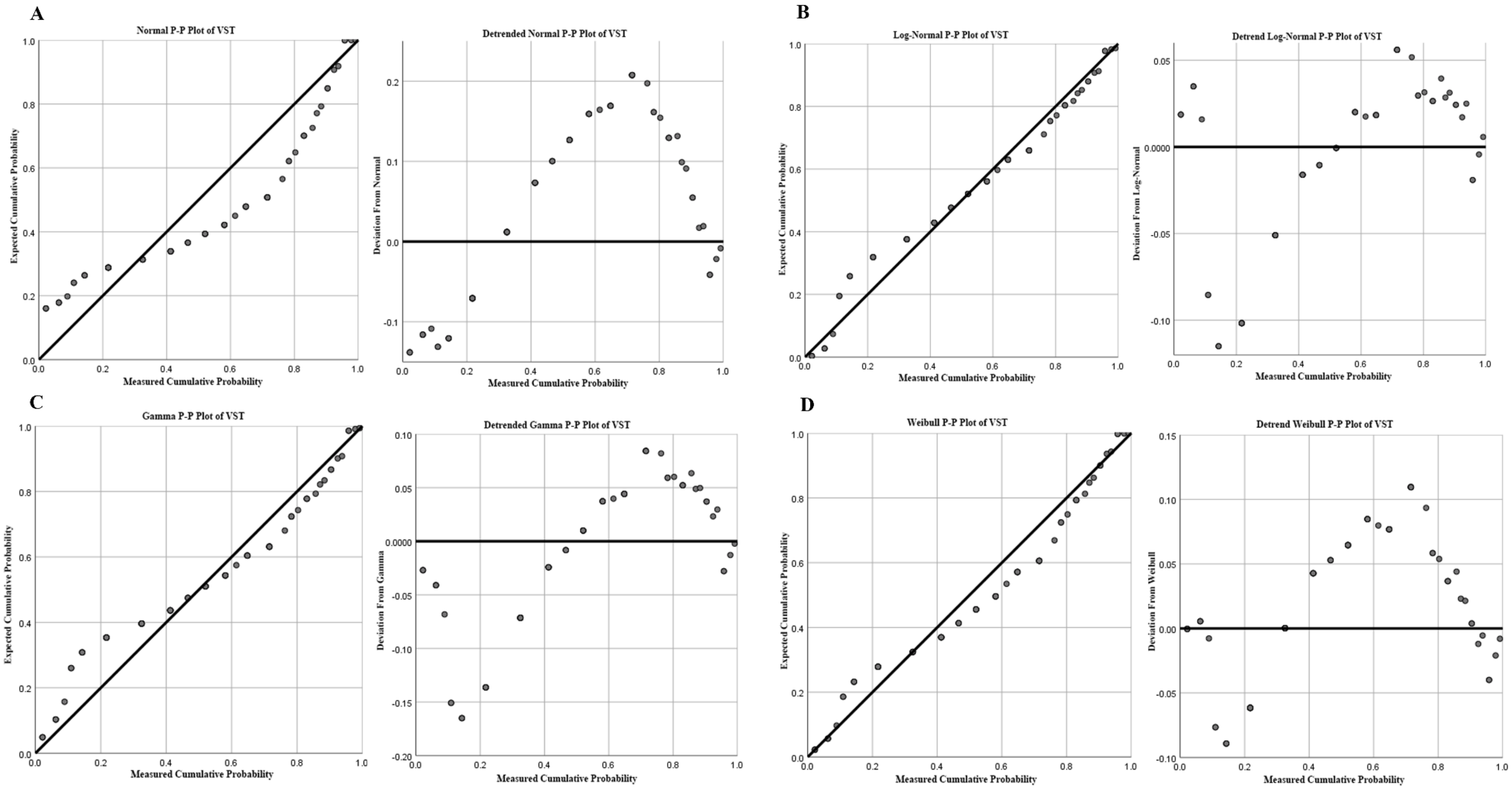

Supplementary figure 1. The P-P plots of viral shedding time of SARS-CoV-2

The individual VSTs of 74 persons infected with SARS-COV-2 were extracted from 6 published articles (1,3,7,14,19,21). Compared with the normal distribution, Weibull distribution and Gamma distribution, we found that the distribution type of the VST was approximately in accordance with the log-normal distribution by using the P-P plots.

References

1. C. Jiehao, X. Jin, L. Daojiong, Y. Zhi, X. Lei, Q. Zhenghai, et al., A Case Series of Children With 2019 Novel Coronavirus Infection: Clinical and Epidemiological Features. Clinical infectious diseases : an official publication of the Infectious Diseases Society of America 71 (2020) 1547-1551. doi: 10.1093/cid/ciaa198.
2. X. Xiong, G.T. Chua, S. Chi, M.Y.W. Kwan, W.H. Sang Wong, A. Zhou, et al., A Comparison Between Chinese Children Infected with Coronavirus Disease-2019 and with Severe Acute Respiratory Syndrome 2003. The Journal of pediatrics 224 (2020) 30-36. doi: 10.1016/j.jpeds.2020.06.041.
3. M.C. Yang, P.P. Hung, Y.K. Wu, M.Y. Peng, Y.C. Chao, and W.L. Su, A three-generation family cluster with COVID-19 infection: should quarantine be prolonged? Public health 185 (2020) 31-33. doi: 10.1016/j.puhe.2020.05.043.
4. J.Y. Noh, J.G. Yoon, H. Seong, W.S. Choi, J.W. Sohn, H.J. Cheong, et al., Asymptomatic infection and atypical manifestations of COVID-19: Comparison of viral shedding duration. J Infect (2020). doi: 10.1016/j.jinf.2020.05.035.
5. T. Zheng, C. Yang, H.-Y. Wang, X. Chen, L. Yu, Z.-L. Wu, et al., Clinical characteristics and outcomes of COVID-19 patients with gastrointestinal symptoms admitted to Jianghan Fangcang Shelter Hospital in Wuhan, China. Journal of Medical Virology 92 (2020) 2735-2741. doi: 10.1002/jmv.26146.
6. S. Lee, T. Kim, E. Lee, C. Lee, H. Kim, H. Rhee, et al., Clinical Course and Molecular Viral Shedding Among Asymptomatic and Symptomatic Patients With SARS-CoV-2 Infection in a Community Treatment Center in the Republic of Korea. JAMA internal medicine (2020). doi: 10.1001/jamainternmed.2020.3862.
7. W. Song, J. Li, N. Zou, W. Guan, J. Pan, and W. Xu, Clinical features of pediatric patients with coronavirus disease (COVID-19). J Clin Virol 127 (2020) 104377. doi: 10.1016/j.jcv.2020.104377.
8. J. Chen, T. Qi, L. Liu, Y. Ling, Z. Qian, T. Li, et al., Clinical progression of patients with COVID-19 in Shanghai, China. J Infect 80 (2020) e1-e6. doi: 10.1016/j.jinf.2020.03.004.
9. L. Zhu, N. Gong, B. Liu, X. Lu, D. Chen, S. Chen, et al., Coronavirus Disease 2019 Pneumonia in Immunosuppressed Renal Transplant Recipients: A Summary of 10 Confirmed Cases in Wuhan, China. European urology 77 (2020) 748-754. doi: 10.1016/j.eururo.2020.03.039.
10. C. Han, C. Duan, S. Zhang, B. Spiegel, H. Shi, W. Wang, et al., Digestive Symptoms in COVID-19 Patients With Mild Disease Severity: Clinical Presentation, Stool Viral RNA Testing, and Outcomes. The American journal of gastroenterology 115 (2020) 916-923. doi: 10.14309/ajg.0000000000000664.
11. X. Yan, X. Han, Y. Fan, Z. Fang, D. Long, and Y. Zhu, Duration of SARS-CoV-2 viral RNA in asymptomatic carriers. Critical care (London, England) 24 (2020) 245. doi: 10.1186/s13054-020-02952-0.
12. Y. Gong, L. Guan, Z. Jin, S. Chen, G. Xiang, and B. Gao, Effects of methylprednisolone use on viral genomic nucleic acid negative conversion and CT imaging lesion absorption in COVID-19 patients under 50 years old. Journal of medical virology (2020). doi: 10.1002/jmv.26052.
13. Y. Warabi, S. Tobisawa, T. Kawazoe, A. Murayama, R. Norioka, R. Morishima, et al., Effects of oral care on prolonged viral shedding in coronavirus disease 2019 (COVID-19). Special care in dentistry : official publication of the American Association of Hospital Dentists, the Academy

of Dentistry for the Handicapped, and the American Society for Geriatric Dentistry (2020). doi: 10.1111/scd.12498.
14. Y. Pan, X. Yu, X. Du, Q. Li, X. Li, T. Qin, et al., Epidemiological and Clinical Characteristics of 26 Asymptomatic Severe Acute Respiratory Syndrome Coronavirus 2 Carriers. J Infect Dis 221 (2020) 1940-1947. doi: 10.1093/infdis/jiaa205.
15. C.Z. Hua, Z.P. Miao, J.S. Zheng, Q. Huang, Q.F. Sun, H.P. Lu, et al., Epidemiological features and viral shedding in children with SARS-CoV-2 infection. Journal of medical virology (2020). doi: 10.1002/jmv.26180.
16. E. Cano, C. Corsini Campioli, and J.C. O'Horo, Nasopharyngeal SARS-CoV-2 viral RNA shedding in patients with diabetes mellitus. Endocrine (2020) 1-2. doi: 10.1007/s12020-020-02516-w.
17. Y. Wu, C. Guo, L. Tang, Z. Hong, J. Zhou, X. Dong, et al., Prolonged presence of SARS-CoV-2 viral RNA in faecal samples. The lancet. Gastroenterology & hepatology 5 (2020) 434-435. doi: 10.1016/s2468-1253(20)30083-2.
18. S. Otsubo, Y. Aoyama, K. Kinoshita, T. Goto, Y. Otsubo, D. Kamano, et al., Prolonged shedding of SARS-CoV-2 in COVID-19 infected hemodialysis patients. Therapeutic apheresis and dialysis : official peer-reviewed journal of the International Society for Apheresis, the Japanese Society for Apheresis, the Japanese Society for Dialysis Therapy (2020). doi: 10.1111/1744-9987.13566.
19. F. Tan, K. Wang, J. Liu, D. Liu, J. Luo, and R. Zhou, Viral Transmission and Clinical Features in Asymptomatic Carriers of SARS-CoV-2 in Wuhan, China. Frontiers in medicine 7 (2020) 547. doi: 10.3389/fmed.2020.00547.
20. T. Xiao, Y. Wang, J. Yuan, H. Ye, L. Wei, X. Liao, et al., Early viral clearance and antibody kinetics of COVID-19 among asymptomatic carriers. (2020). doi: 10.1101/2020.04.28.20083139.
21. Y. XY, Familial clustering infection caused by asymptomatic COVID-19 (in Chinese). Int J Respi 40 (2020).
22. Y.J. Liu, P. Chen, Z.S. Liu, Y. Li, H. Du, and J.L. Xu, Clinical features of asymptomatic or subclinical COVID-19 in children (in Chinese). Zhongguo dang dai er ke za zhi = Chinese journal of contemporary pediatrics 22 (2020) 578-582. doi: 10.7499/j.issn.1008-8830.2004088.
23. S. ZY, H. YH, Y. M, D. M, W. Y, L. M, et al., Influencing factors of negative conversion time of viral nucleic acid of nasopharyngeal swabs in patients with novel coronavirus infected pneumonia (in Chinese). Practical Journal of Clinical Medicine 17 (2020) 45-48.
24. L. L, Y. HS, G. CY, W. XJ, L. XL, Z. JY, et al., Traditional Chinese medicine characteristics of 46 patients with COVID-19 (in Chinese). Journal of Capital Medical University 41 (2020) 155-160.
25. J. Y, W. HZ, S. G, D. LJ, O. CR, and R. XM, Clinical ananlysis of corona virus disease 2019: a report of 24 cases (in Chinese). Journal of Chongqing Medical University 45 (2020) 968-970.
26. G. HL, H. HP, Z. X, L. SS, Z. ZH, Z. SF, et al., Related Factors of the Conversion Time of Virus Nucleic Acid Turning Negative in Patients with Coronavirus Disease 2019 and Its Effect on Prognosis (in Chinese). Herald of Medicine 39 (2020) 811-814.
27. Z. BN, L. DF, L. YL, L. LJ, D. Q, C. H, et al., Differences in Laboratory Indicators and Prognosis Between COVID-19 Patients with or without Diabetes Mellitus (in Chinese). Sichuan Medical Journal 41 (2020) 913-917.

28. Z. BY, Z. ZY, L. ZH, C. Q, Z. JY, and D. XJ, Retrospective analysis of viral nucleic acid test results of 116 fecal samples from COVID-19 patients. Jinagsu Medical Journal 46 (2020) 571-572.
29. X. LX, Clinical characteristics of 59 cases of COVID-19 in Guangzhou (in Chinese). China Tropical Medicine 20 (2020).
30. X. CM, Q. P, Z. XP, D. HB, C. SJ, and X. YX, Dynamic changes of IgM and IgG in confirmed COVID-19 cases: detection results analysis (in Chinese). Chin J Publ Heal 36 (2020) 1396-1398.
31. S. LL, L. XD, L. SX, N. CZ, P. SG, W. QL, et al., Nucleic acid detection analysis of 71 cases of COVID-19 with biological samples of diferent disease course(in Chinese). China Tropical Medicine 20 (2020) 763-766.
32. R. HJ, L. H, S. GX, L. H, B. JW, and T. LX, Clinical characteristics of 2019 novel coronavirus pneumonia in a mobile cabin hospital in Wuhan: analysis of 94 cases (in Chinese). Chinese Journal of Hygiene Rescue 6 (2020) 209-213.
33. R. QH, C. H, Z. L, and Z. YC, Analysis of Factors Influencing Viral Shedding Time in Severe and Critical COVID-19 Patients (in Chinese). Advances in Clinical Medicine 10 (2020) 1717-1724. doi: 10.12677/acm.2020.108258.
34. L. JJ, Influencing factors for positive duration of viral nucleic acid in patients with severe coronavirus disease 2019 (in Chinese). Academic Journal of Second Military Medical University 41 (2020).
35. L. N, X. T, W. XF, Y. SY, C. YQ, J. SY, et al., Chloroquine phosphate accelerates the conversion of nucleic acid to negative in 88 common COVID-19 patients (in Chinese). The Journal of Practical Medicine (2020) 1-4.
